# Supplementary material for: Comprehensive Analysis of Differentially Expressed CircRNAs in the Ovaries of Low- and High-Fertility Sheep
Source: Animals (Basel). 2023 Jan 9;13(2):236. doi: 10.3390/ani13020236 (PMC9854751; doi:10.3390/ani13020236)
Supplement: Supplementary file 1 [file animals-13-00236-s001.zip › Supplementary Figure.pdf]

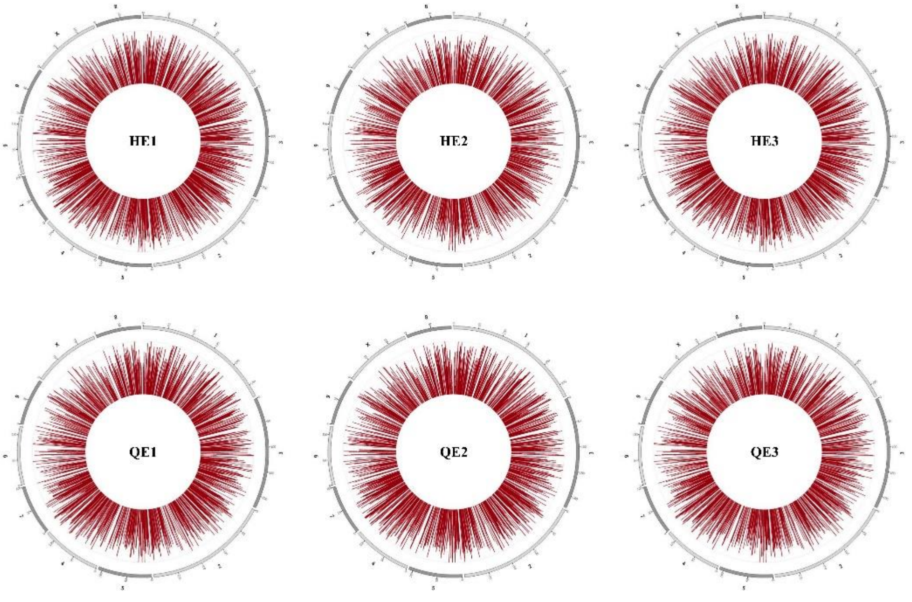

Supplementary Figure S1. Density distribution of circRNA on QE and HE chromosomes.

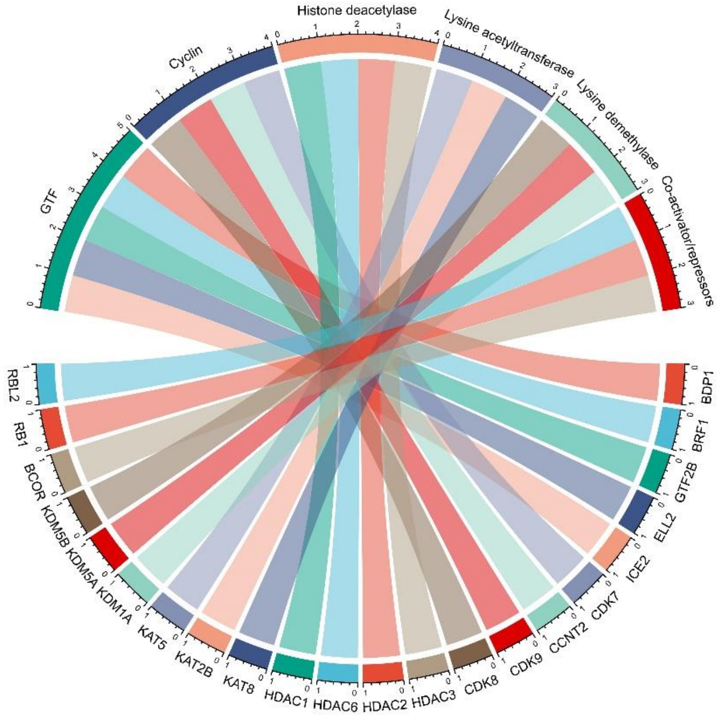

Supplementary Figure S2. Top 5 family statistics of TF predictions for the three genes.

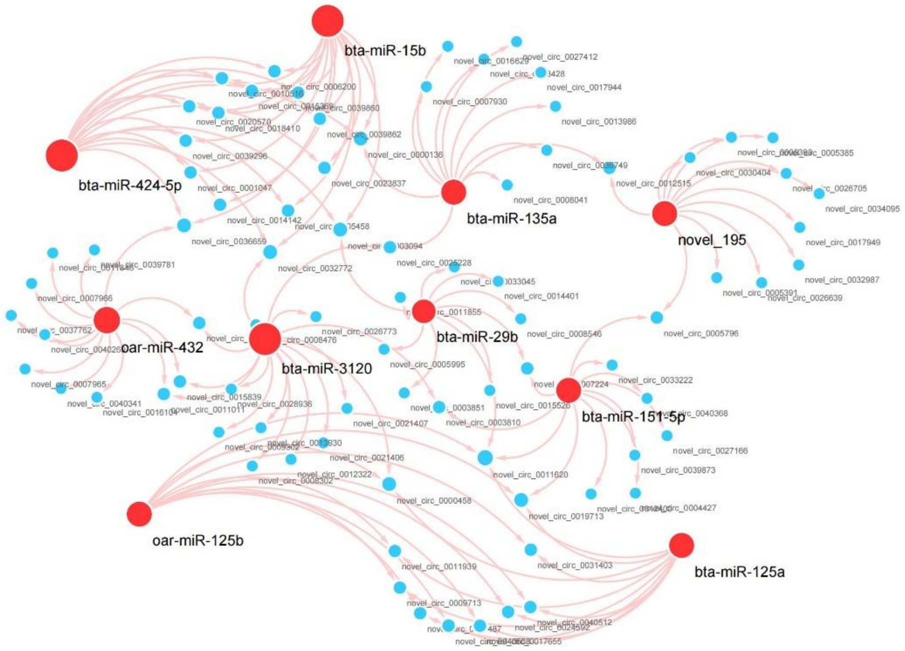

Supplementary Figure S3. The top 10 miRNAs with the most nodes in the circRNA-miRNA regulatory network. red circles represent miRNAs and dark blue circles represent miRNAs.

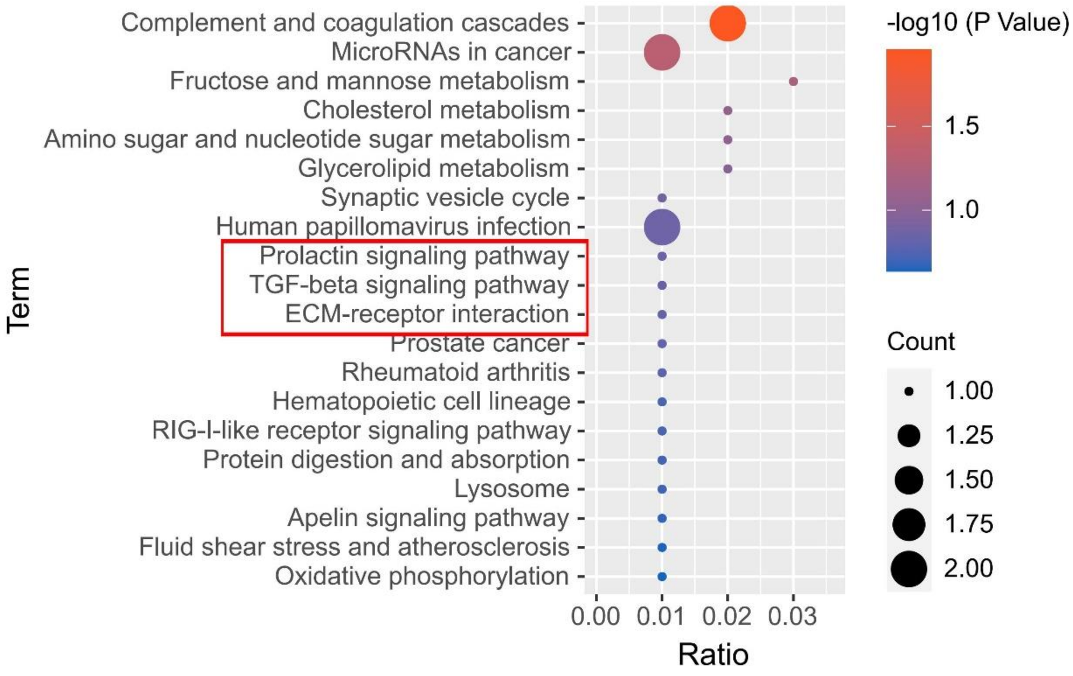

Supplementary Figure S4. KEGG enrichment analysis of DEmiRNA target DEmRNAs.

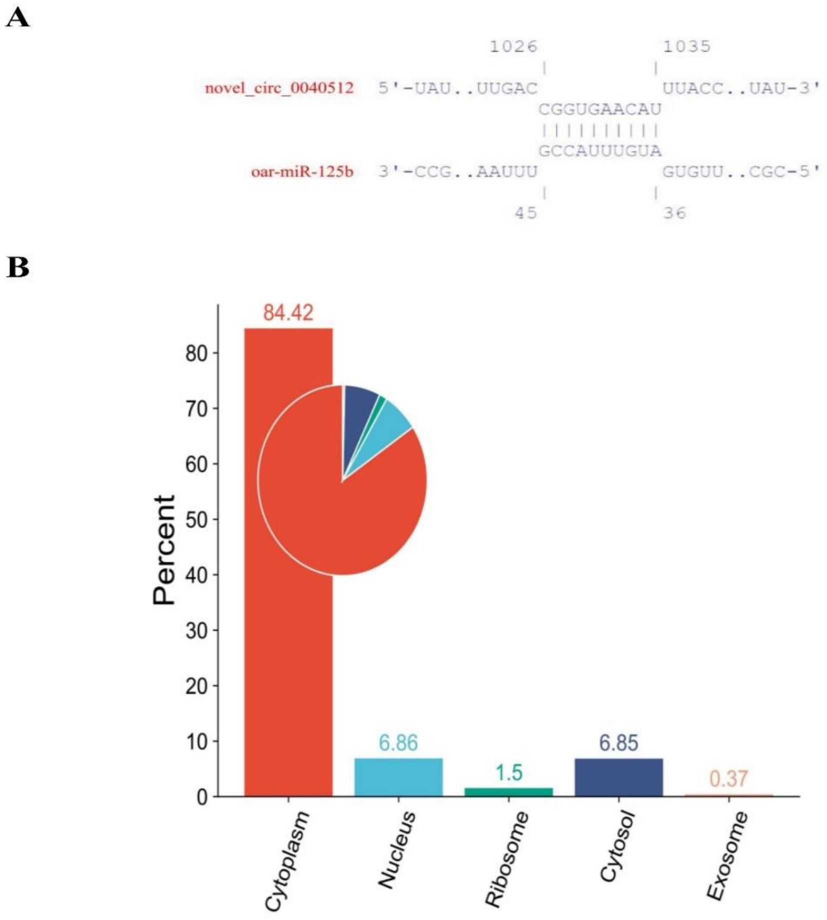

Supplementary Figure S5. Novel-circ-0040512 sponge oar-miR-125b in sheep ovarian granulosa cells. (A) Predicted circRNA shear site and oar-miR-125b complementary binding site. (B) Predicted distribution of novel-circ-0040512 in cells.
